# Effect of Liquid Nitrogen Freezing on Maintaining the Quality of Crayfish During Freeze–Thaw Cycles: Muscle Structure and Myofibrillar Proteins Properties

**DOI:** 10.3390/foods14020279

**Published:** 2025-01-16

**Authors:** Zongna Teng, Xiaoyue He, Liuqing Wang, Limin Xu, Chuyi Jiao, Jiwang Chen

**Affiliations:** 1 College of Food Science and Engineering, Wuhan Polytechnic University, Wuhan 430023, China; t2806710332@163.com (Z.T.); hxiaoyue2024@163.com (X.H.); lm001011@163.com (L.X.); 2 Hubei He Yuan Gas Co., Ltd., Yichang 443000, China; 18672667710@163.com; 3 Hubei Key Laboratory for Processing and Transformation of Agricultural Products, Wuhan Polytechnic University, Wuhan 430023, China

**Keywords:** liquid nitrogen freezing, freeze–thaw cycle, myofibrillar proteins, drip loss, ice crystal

## Abstract

The quality of frozen crayfish (*Procambarus clarkii*) is challenged by freeze–thaw (FT) cycles during storage. The effect of freezing methods on the quality of crayfish during FT cycles was investigated by comparing physicochemical properties, microstructure, and myofibrillar protein (MPs) properties. Three methods were used for crayfish freezing, including air convective freezing (AF) at −20 °C and −50 °C, as well as liquid nitrogen freezing (LNF) at −80 °C. The frozen crayfish were thawed at 4 °C after 45 d of frozen storage as 1 FT cycle. After 5 FT cycles, the water holding capacity of LNF crayfish (70.8%) was significantly (*p* < 0.05) higher than that of −20 °C AF crayfish (60.6%) and −50 °C AF crayfish (63.5%). The drip loss of LNF crayfish (7.83%) was significantly lower than that of AF crayfish. Moreover, LNF maintained the gel strength and the thermal stability of MPs from crayfish with higher gel storage modulus and enthalpy. These results demonstrated that LNF minimized the formation of large ice crystals, preserving the structural integrity of muscle and the properties of MPs, thereby maintaining crayfish quality. This study investigated the effect of LNF in preserving crayfish quality during FT cycles, providing valuable insights for reducing the quality degradation of aquatic products during storage and transportation.

## 1. Introduction

Crayfish (*Procambarus clarkii*) is a vital economic freshwater product extensively spread throughout the Yangtze River basin in China, which enjoys customer preference because of its unique flavor, premium proteins, and low fat content [1]. Crayfish Market Report and Forecast reported that the global crayfish market is expected to grow by 31.5% between 2024 and 2032, reaching about $171.68 billion in 2032 [2]. The total output value of China’s crayfish industry reached 64.07 billion in 2022, marking an annual growth of 7.99% above the 2021 level of 59.33 billion [3]. Freshness is an important factor influencing the quality of crayfish [4]. However, the limitation of seasonal and regional characteristics of crayfish production will hinder the industry process and sales [5]. Crayfish is susceptible to spoilage and deterioration from oxidation reactions, microbial growth, and enzymatic reactions owing to its high water content, leading to a decrease in both quality and market value [6]. Therefore, the preservation of crayfish during storage and transportation has important economic value in maintaining its premium quality and promoting its high-quality development [7].

Freezing is the most common and conventional technique used to maintain the quality of crayfish [8]. In the freezing process, the conversion of free water in crayfish into ice crystals reduces microbial growth, as well as chemical and enzymatic reactions, thus retaining the original characteristics of crayfish [9]. However, freeze–thaw (FT) cycles frequently arise from deficiencies in cold chain logistics, while the temperature fluctuation will aggravate the generation of large ice crystals and recrystallization in crayfish muscle [10]. For the muscle structure of crayfish, myofibrillar proteins (MPs) are the uppermost protein component and are responsible for the muscle’s texture and functional properties [11]. Research of Lee et al. [12] has shown that the extracellular ice crystallization and the shift of intracellular solute concentrations accelerated the freezing-induced denaturation of MPs, resulting in the severe spoilage of frozen foods. Therefore, the characteristics of ice crystals should impact the muscle structure and MPs properties, ultimately affecting the quality of frozen crayfish during FT cycles.

There are many measures used to maintain the quality of frozen foods during FT cycles, including quick freezing, the addition of cryoprotective agents, and the application of physical electric field forces [13]. Among these methods, the optimization of freezing methods is a green, safe, and cost-effective approach without additives and additional procedures. Air convective freezing (AF) is the most conventional freezing method and requires a long freezing time due to the poor thermal conductivity of air medium. Liquid nitrogen freezing (LNF) is an effective freezing technology for the preservation of aquatic products [11]. The freezing rate of LNF is higher than that of AF owing to the higher heat transfer coefficient of liquid nitrogen, enabling LNF to form small ice crystals within and across the muscle fibers, thereby reducing mechanical damage to the cell structure during the freezing process [14]. Therefore, LNF may effectively maintain the integrity of cells and muscle structure compared to other freezing methods. Teng et al. [15] reported that LNF could effectively reduce the freezing time and promote the freezing rate compared to AF, and the freezing time was significantly shorter (*p* < 0.05) as the liquid nitrogen temperature decreased. Huang et al. [6] investigated the preservation mechanism of LNF on crayfish and confirmed that LNF was a promising freezing process for maintaining the biochemical and structural properties of fresh crayfish, including less protein denaturation, better texture, and more uniform muscle fibers.

The current studies have confirmed that LNF could maintain the quality of frozen crayfish, as well as protect the muscle structure and protein characteristics. It can be inferred that LNF may be a feasible solution for the quality preservation of crayfish from the damage of ice crystals during FT cycles. However, the effects of freezing methods on the crayfish quality (muscle structure and MPs properties) during FT cycles are not clear, which will hinder the development of the frozen crayfish industry. To investigate the effect of LNF on the quality maintenance of crayfish during FT cycles, three conditions were taken for crayfish freezing (AF at −20 °C, AF at −50 °C, and LNF at −80 °C). The properties of frozen crayfish during 1 to 5 FT cycles were measured and evaluated compared with non-frozen crayfish. Through multivariate statistical analysis of the physicochemical properties and MPs characteristics of crayfish, the effects of LNF on the quality maintenance of crayfish during FT cycles were explained. The expectation for these studies is to understand changes in the muscle structure and the MPs properties of crayfish by different freezing methods during FT cycles and investigate the theoretical basis of LNF on quality maintenance of crayfish during FT cycles. This will promote the refinement and expansion of the crayfish market.

## 2. Materials and Methods

### 2.1. Materials and Reagents

Hydrochloric acid standard titration solution (0.01 M) was from Shenzhen Berlinda Technology Co., Ltd. (Shenzhen, China). Glutaraldehyde fixative (2.5%, *w*/*w*) was purchased from Shanghai Yuan ye Biotechnology Co., Ltd. (Shanghai, China). Methane (Tris) was from Bio froxx GmbH (Einhausen, HE, Germany). Additional analytical-grade reagents and chemicals were purchased from Sinopharm Chemical Reagent Co., Ltd. (Shanghai, China).

### 2.2. Sample Preparation

Alive adult crayfish were purchased from Hubei Qianwang Ecological Crayfish Industrial Park Group Co., Ltd. (Qianjiang, China). and transported to the laboratory within 48 h in a foam box with ice packs at a temperature of 0–4 °C to ensure freshness. Upon arrival, healthy, active crayfish weighing 20.0 ± 5.0 g and exhibiting a bright red color were selected for the study. The crayfish were immersed in an ice water bath for 60 min to induce unconsciousness. The selected crayfish were then scalded after cleaning thoroughly [1,16]. The non-frozen crayfish were used as the control group, named NF, and were immediately processed for analysis. The remaining crayfish were distributed into the three groups randomly. Two groups were frozen in BD/BC-301KM(E) refrigerator (Hefei Midea Refrigerator Co., Ltd., Hefei, China) at −20 °C and −50 °C, named −20 °C AF and −50 °C AF, respectively. A group of crayfish was frozen by a liquid nitrogen cabinet (Cryogenic (Beijing) Science & Technology Co., Ltd., Beijing, China) at −80 °C [15], named LNF. The three groups of crayfish were frozen to a central temperature of −18 °C and transferred to a −18 °C refrigerator for 45 d. Then these crayfish were thawed at 4 °C for 24 h, which was the 1 FT cycle. The thawed crayfish were frozen again by −20 °C AF, −50 °C AF, and LNF, respectively, then were stored at −18 °C refrigerator and thawed after 45 d, which was the 2 FT cycles. The thawed crayfish and the NF crayfish were removed head, shell, and intestinal line for analysis. According to the researches about crayfish during FT cycles, five times of FT cycles were taken in this study [16,17]. Crayfish tails after completing 1, 2, 3, 4, and 5 FT cycles were taken for analysis, named 1 FT, 2 FT, 3 FT, 4 FT, and 5 FT, respectively.

### 2.3. Color Difference Analysis

The color of the second abdominal segment of the crayfish was assessed based on Alizadeh-Sani et al. [18] using a JZ-300 portable color meter (Shenzhen Jinjun Instrument and Equipment Co., Ltd., Shenzhen, China), which was warmed up for 30 min and calibrated with a whiteboard. The L*, a*, and b* values were recorded after wiping away the surface water from the thawed crayfish with filter paper. The total color difference values (Δ*E*) were used to determine the degree of decay in contrast to fresh crayfish. Δ*E* was calculated using Equation (1):

(1) $$\Delta E=\sqrt{{\left(L_{0}*-L*\right)}^{2}+{\left(a_{0}*-a*\right)}^{2}+{\left(b_{0}*-b*\right)}^{2}}$$

where L*, a*, and b* were the lightness/darkness, redness/greenness, and yellowness/blueness values of crayfish, respectively. *L*_0_*, *a*_0_*, and *b*_0_* represent the value of NF.

### 2.4. Measurement of pH, Total Volatile Basic Nitrogen (TVB-N), Drip Loss (DL), Water Holding Capacity (WHC), Shear Force

#### 2.4.1. pH

The pH of the crayfish was determined based on Yu et al. [19] with minor modifications. The weight of 2 g crayfish tails was crushed, then homogenized with 20.0 mL distilled water using an XHF-DY high-speed homogenizer (Ningbo Scientz Biotechnology Co., Ltd., Ningbo, China) at a speed of 2000 rpm. The pH values of the homogenate were measured using a DELTA-320pH meter (Mettler-Toledo International Trading Co., Ltd., Shanghai, China). The results are expressed as the mean pH value ± standard deviation.

#### 2.4.2. TVB-N

The TVB-N value of the crayfish was analyzed by the semi-micro steam distillation method according to Ruan et al. [20] with minor modifications. The crayfish tails were accurately weighed 5 g into a 50 mL centrifuge tube with 25 mL trichloroacetic acid solution (20 g/L). The mixture was homogenized using an XHF-DY high-speed homogenizer (8000 rpm) in an ice bath for 10 s, and the filtrate was collected. The supernatant (5 mL) was added with the volume of 5 mL 30 g/L NaOH and the phenolphthalein indicator, and distilled using a semi-micro nitrogen fixing device (Jinan Bomei Experimental Equipment Co., Ltd., Jinan, China). The distillate was collected in the bottle containing 10 mL of 30 g/L boric acid solution with a mixed indicator of methyl red and bromocresol green. The mixture then was titrated with a 0.01 M hydrochloric acid standard solution. The TVB-N value was obtained by conversion of the volume of titrated hydrochloric acid. The blank was also carried out with 10 mL of trichloroacetic acid solution. The results are expressed in milligrams of nitrogen per 100 g of sample (mg N/100 g) ± standard deviation.

#### 2.4.3. DL

The DL of the crayfish was determined based on Li et al. [21]. The weight of crayfish tails was initially measured before thawing (*m*_1_). After thawing at 4 °C for 24 h, the samples were dried with absorbent paper and weighed once more (*m*_2_). DL was determined by Equation (2):

(2) $$DL\left(\%\right)=\frac{m_{1}-m_{2}}{m_{1}}\times 100$$

where *m*_1_ and *m*_2_ represent the mass of crayfish tails before and after thawing, respectively; 100 represents the conversion factor.

#### 2.4.4. WHC

The WHC of the crayfish was determined based on Bao et al. [22]. Crushed crayfish tails weighing *m*_3_ were set into a centrifuge tube with a bottom pre-lined quantified filter paper weighing *m*_4_. The tube was centrifuged in a TGL-16C centrifuge (Shanghai Anting Scientific Instrument Factory, Shanghai, China) at 5000 rpm for 10 min. After separating the crayfish tails from the filter paper, the weight of the filter paper was recorded as *m*_5_. The WHC was calculated using Equation (3).

(3) $$\mathrm{WHC}\left(\%\right)=1-\frac{m_{5}-m_{4}}{m_{3}}\times 100$$

where *m*_3_ represents the mass of crayfish meat, g; *m*_4_ represents the mass of the filter paper before centrifugation, g; *m*_5_ represents the mass of the filter paper after centrifugation; and 100 represents the conversion factor.

#### 2.4.5. Shear Force

The measurement of the shear force referred to the method of Du et al. [23]. The blocks (1.2 cm × 0.5 cm × 0.5 cm) from the crayfish tails were placed on the blade of the C-LM3B digital muscle tenderizer (College of Engineering, Tohoku University of Agriculture, Tokyo, Japan) with the knife edge perpendicular to the direction of the fiber. The crosshead speed of the knife blade was 5 mm/s. The results are expressed in Newtons (N) ± standard deviation.

### 2.5. Observation of Ice Crystal Microstructure

The observation of ice crystals in the microstructure of crayfish was determined according to Han et al. [7]. The crayfish tails with the size of 1 mm × 1 mm × 1 mm were set into a mixture of 60% absolute ethanol, 30% chloroform, and 10% glacial acetic acid. The mixture was frozen at −18 °C for 18 h, then heated to 25 °C. The crayfish tails were dehydrated with absolute ethanol once, n-butanol solution twice for 2 h each time, and finally soaked in n-butanol solution for more than 8 h. After dehydration, the blocks were washed with toluene for 30 min, then soaked in liquid paraffin at 57 °C three times, each lasting 1 h. The blocks were fixed in paraffin wax with a mold and trimmed into sections with a thickness of 10 μm along with the muscle fiber orientation. The sections with water were placed on a 56 °C plate to melt the paraffin, then immersed in toluene twice and absolute ethanol twice for 10 min each time. Finally, the tissue was sealed after being stained with bright green solution for 3 min, and washed with absolute ethanol and toluene solution 3 times, respectively. Afterward, the tissue sections were observed and captured using a light microscope (Nikon Instruments Inc., New York, NY, USA). The average area of ice crystals in muscle fibers was calculated from a minimum of three images of frozen crayfish, and the number of valid ice crystals in the images was not less than 100 to ensure the accuracy of data analysis [24]. The images were processed with ImageJ software v. 1.53e (ImageJ, Bethesda, MD, USA). The results are expressed as the average area of ice crystals in square micrometers (μm^2^) ± standard deviation.

### 2.6. Scanning Electron Microscopy (SEM)

The abdominal segment of crayfish tails was sectioned into 1 mm^3^ cubes and then fixed in 4% glutaraldehyde solution for 12 h. After a sequential dehydration process with ethanol solutions of 50%, 70%, 80%, 90%, and 100% ethanol solutions, the crayfish tails were dried in a freezing dryer (Beijing Boyikang Experimental Instrument Co., Ltd., Beijing, China), then sputter-coated with gold for subsequent observation. Afterward, the microstructure was observed using a TM4000 PLUS II scanning electron microscopy (SEM, Hitachi High-Technologies Co., Ltd., Beijing, China) according to the method of Mi et al. [25].

### 2.7. Extraction of Myofibrillar Proteins from Crayfish

The extraction of MPs from crayfish tails followed the method of Zhang et al. [26] with some modifications. The crayfish tails of 3 g were homogenized in 30 mL extraction solution A (1:10, *w*/*v*), comprising 0.1 M KCl and 20 mM Tris-maleate buffer with a pH of 7.5. The homogenate was centrifuged at 10,000 rpm at 4 °C for 20 min to obtain the precipitate. The precipitate was rinsed with 30 mL extraction solution B comprising 0.6 M KCl and 20 mM Tris-maleate buffer with a pH of 7.0, then homogenized for 2 min. After 60 min of extraction at 4 °C, the homogenate was centrifuged to get the supernatant as MPs solution for analysis. The supernatant was regarded as soluble MPs.

### 2.8. Measurement of Rheological Properties of MPs

The rheological properties of MPs were measured using a Rheometer (Kinexus pro+, New Castle, DE, USA). The rheometer was set to oscillation mode, maintaining a linear range at a frequency of 0.1 Hz and a strain of 0.5% for shearing. The volume of 1 mL MPs solution was added into the stage of the rheometer with the silicone oil to prevent water evaporation. MPs were maintained at 25 °C for 3 min, followed by a gradual increase to 80 °C at a heating rate of 2 °C/min. During heating, the rheological curves of MPs were determined. The results are expressed as the storage modulus (*G*′) at different temperatures.

### 2.9. Measurement of Thermal Stability of MPs

The thermal properties of crayfish were determined through differential scanning calorimetry (DSC, Q-2000, TA Instruments, New Castle, DE, USA) based on Yang et al. [9]. The crayfish tails of 5 mg were deposited into an aluminum container, sealed, and heated from 20 °C to 80 °C at a rate of 5 °C/min. Enthalpy (Δ*H*) and denaturation temperature (*T_max_*) were estimated from the thermogram by Pyris software v.11.0 (TA Instruments, New Castle, DE, USA). The results are expressed as the *T_max_* and Δ*H* for myosin and actin.

### 2.10. Statistical Analysis

The experiment was conducted in triplicate. For repeated experiments, the results were depicted as mean and standard deviation. The data were analyzed using one-way analysis of variance (ANOVA), and the significant difference between the means was identified using Duncan’s multiple range test (*p* < 0.05), facilitated by SPSS statistics software v.19.0 (IBM, Armonk, NY, USA) [17]. The PCA analysis was conducted using Origin 2023 (Origin Lab Corporation, Northampton, MA, USA).

## 3. Results and Discussion

### 3.1. Color Analysis

**Figure 1:**
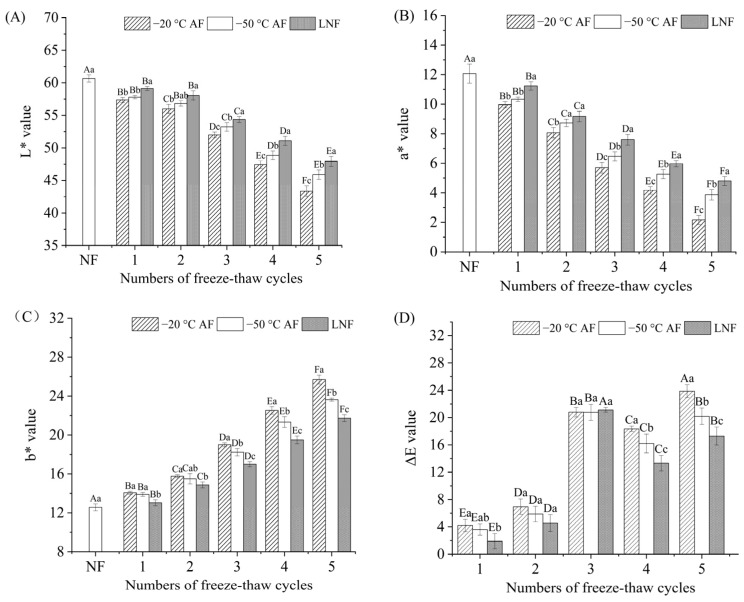
Changes in color of crayfish during freeze–thaw cycles. (**A**) L* value; (**B**) a* value; (**C**) b* value; (**D**) Δ*E* value. NF represents non-frozen crayfish, AF represents air convective freezing, and LNF represents −80 °C liquid nitrogen freezing. The labels 1, 2, 3, 4, 5 FT represent frozen crayfish with 1, 2, 3, 4, 5 freeze–thaw cycles. Notes: Different letters in the same column indicate a significant difference (*p* < 0.05). Lowercase letters indicate significant differences among freezing methods, and capital letters indicate significant differences among the number of freeze–thaw cycles.

The color of meat is a crucial attribute that significantly impacts its marketability and consumers’ willingness to purchase [27]. The color of crayfish subjected to three different freezing methods during FT cycles was shown in Figure 1. The results showed that the L* and a* values of three crayfish groups significantly (*p* < 0.05) declined with the increasing number of FT cycles. In contrast, the b* and Δ*E* values were significantly increased. This result was consistent with the color analysis of *Sardinella aurita* during FT cycles reported by Megaache et al. [28]. The observed decline in the L* values of the three crayfish groups during FT cycles may be attributed to the movement of water from the interior of myofibrils to the spaces between them, ultimately emerging on the crayfish surface as drip [29]. This result aligns with the research conducted by Liu et al. [10], which noted similar effects of water movement due to temperature variations. The decline in the a* values of the three crayfish groups during FT cycles could be attributed to the oxidative denaturation of proteins. Li et al. [30] discovered a notable drop in the a* value of frozen pork, suggesting that this decline could be attributed to the breakdown of myoglobin and the creation of hyper myoglobin during the freezing process. Additionally, lipid oxidation can induce free radicals, which accelerate the oxidation of myoglobin. Sun et al. [31] have reported that the increase in the b* values of crayfish during FT cycles was caused by lipid oxidation, which worked in conjunction with protein oxidation to create a yellower color of crayfish. The Δ*E* value reflected the extent of the color change of muscle, and the increase of Δ*E* value was closely connected with the deterioration extent of crayfish. For AF groups, the L* and a* values of −50 °C AF crayfish were higher than those of −20 °C AF crayfish, whereas the b* and Δ*E* values of the −50 °C AF group were lower. This indicated that a lower freezing temperature could better prevent the color change of crayfish during FT cycles. In addition, the L* and a* values of LNF crayfish were significantly higher than those of AF groups, while the b* and Δ*E* values of LNF group were lower. This could be because the LNF with a higher freezing rate could have slowed the chemical reactions within crayfish, including the deoxygenation of myoglobin and the oxidation of lipids [13,32]. Consequently, LNF crayfish exhibited a color closer to that of NF crayfish during FT cycles, thus better maintaining their appearance.

### 3.2. Quality Characteristics Analysis

The quality characteristics of crayfish frozen by three freezing methods during FT cycles were shown in Table 1, including pH, TVB-N, DL, WHC, and shear force.

The pH value serves as an indicator of crayfish freshness, and it is positively correlated with the degree of deterioration. The pH values of the three crayfish groups showed a trend of decline before 3 FT cycles, followed by an increase with the increasing numbers of FT cycles. The initial decline in pH value was due to the degradation of sugars in crayfish into acids before the 3 FT cycle. Subsequently, the endogenous enzymes and/or microorganisms broke down amino acids in crayfish into ammonia and other compounds during freezing and thawing, which further elevated the pH [10]. The pH value of LNF crayfish (8.50) was significantly lower (*p* < 0.05) than that of −20 °C AF crayfish (8.68) and −50 °C AF crayfish (8.59) after 5 FT cycles. Moreover, the pH value of LNF crayfish was closest to that of NF crayfish (7.96) during FT cycles. This finding was consistent with the research conducted by Abdelnaby et al. [33], which noted that the initial pH of cooked crayfish was 7.31 and changed significantly during frozen storage. The pH value of crayfish increased as the frozen storage durations were prolonged. Du et al. [34] investigated the impact of FT cycles on the quality of rainbow trout stored at −20 °C. They found that the decrease in pH was likely due to the breakdown of muscle glycogen during thawing. Additionally, the concentration of solutes increased with the water loss from muscle, leading to protein denaturation, which causes an increase in pH. This demonstrated that LNF could effectively maintain the freshness of crayfish, keeping it closer to NF crayfish during FT cycles.

TVB-N value indicates nitrogenous substances such as ammonia and amines that result from protein decomposition. This measurement is a crucial indicator of freshness when assessing the spoilage level of crayfish [35]. The TVB-N values of three crayfish groups were increased significantly with increasing FT cycle numbers, which might be attributed to the breakdown of proteins and nonprotein nitrogenous substances, as well as the metabolism of spoilage bacteria [36]. Among the three groups, −20 °C AF crayfish exhibited the highest TVB-N value of 24.01 mg/100 g after 5 FT cycles. The −50 °C AF crayfish had a TVB-N value of 20.28 mg/100 g, indicating that a lower temperature of AF could retard the spoilage of frozen crayfish during FT cycles. Moreover, the TVB-N value of the LNF group (18.39 mg/100 g) was significantly lower than that of the AF group, which could be attributed to the inhibition of spoilage microorganisms and endogenous enzyme activity by LNF [6]. Liu et al. [37] found that the TVB-N levels in the refrigerated shrimp group surpassed freshness limits by day 4 and became inedible by day 8. The crayfish subjected to FT cycles maintained freshness until day 8 and remained edible quality until day 21. This indicates that FT may slow the increase in TVB-N more effectively than refrigeration (*p* < 0.05).

The DL and WHC could reflect the ability of muscle to retain water in spatial structure with MPs as the essential constituents, which are important indicators in meat quality assessment [38]. The DL values of the three crayfish groups were significantly increased, while the WHC decreased significantly with the increase of FT cycle numbers. Specifically, the WHC value of LNF crayfish (70.8%) was significantly higher than that of −20 °C AF (60.6%) and −50 °C AF (63.5%) after 5 FT cycles. The DL value of LNF crayfish was increased to 7.83% after 5 FT cycles compared to NF crayfish (2.18%), which was significantly lower than that of AF crayfish. For the FT cycles, the formation of large ice crystals during freezing may cause irreversible damage to the cell membrane, generating drip losses and consequent cellular dehydration during thawing [39]. Meanwhile, the growth of large ice crystals and recrystallization could destroy the muscle tissue, causing the decline of WHC, which could further lead to the increase in DL of crayfish during FT cycles [40]. This conclusion was consistent with the research of Liu et al. [10]. These results demonstrated that LNF would effectively alleviate the damage to the muscle structures, inhibiting drip losses from crayfish muscle during FT cycles.

Shear force is employed to reflect the texture properties of crayfish [41]. Compared to the shear force of NF crayfish (18.3 N), the shear force of −20 °C AF crayfish, −50 °C AF crayfish, and LNF crayfish was decreased to 11.0 N, 11.5 N, and 12.1 N after 5 FT cycles, respectively. Leygonie et al. [42] reported that the decline of shear force was due to the fact that the ice crystals destroyed muscle fibers and cell membranes. Meanwhile, the shear force of LNF crayfish was significantly higher than that of AF crayfish during FT cycles, indicating that LNF could better maintain the integrity of muscle fibers during FT cycles.

Above all, as the number of FT cycles increased, protein and nonprotein nitrogenous substances were degraded due to the activities of microorganisms and endogenous enzymes. This resulted in an increase in pH and TVB-N values of crayfish. On the other hand, larger ice crystals could disrupt the cellular integrity and the muscle structure, resulting in a decrease in WHC and shear force, as well as the increase in DL of crayfish. Among the three freezing methods, LNF can effectively keep the WHC and shear force of muscle and avoid thawed drip losses, thus maintaining the quality of crayfish during FT cycles. Huang et al. [6] have confirmed that LNF could quickly pass the “maximum ice crystal formation zone” owing to its fast freezing speed and strong antioxidant properties. This facilitated the creation of a cold environment and the formation of nucleation, resulting in the stimulation of numerous nucleation points and the generation of smaller ice crystals [43]. Sun et al. [44] demonstrated that the size and uniformity of ice crystals affected the shear force, DL, and microstructure of frozen aquatic products. The ice crystal morphology and muscle structure of crayfish should be observed to explain the effect of LNF on the crayfish quality.

### 3.3. Ice Crystal Morphology and Size Analysis

**Figure 2:**
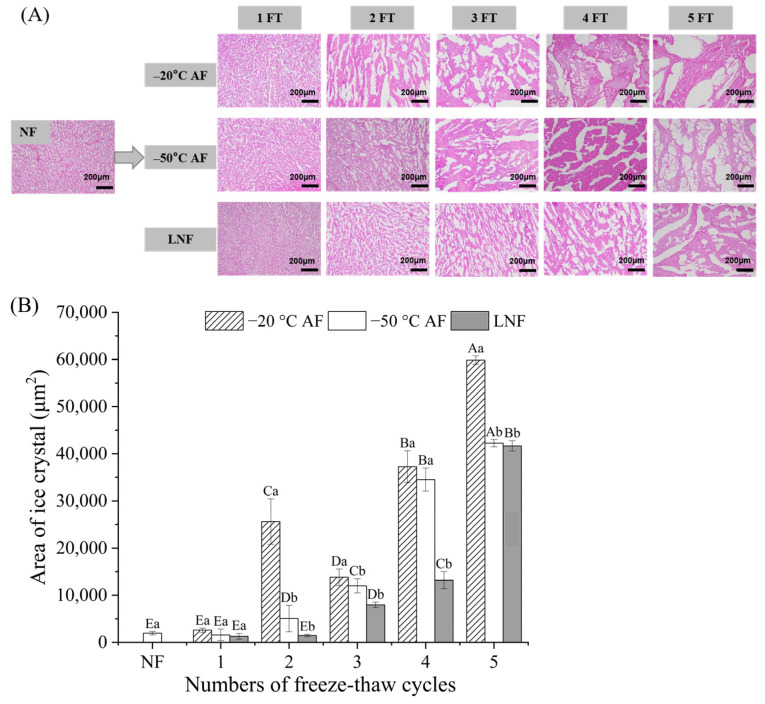
Changes in ice crystal morphology of crayfish during freeze–thaw cycles. (**A**) Ice crystal morphology; (**B**) area of ice crystal. NF represents non-frozen crayfish, AF represents air convective freezing, and LNF represents −80 °C liquid nitrogen freezing. The labels 1, 2, 3, 4, 5 FT represent frozen crayfish with 1, 2, 3, 4, 5 freeze–thaw cycles. Notes: Different letters in the same column indicate a significant difference (*p* < 0.05). Lowercase letters indicate significant differences among freezing methods, and capital letters indicate significant differences among the numbers of freeze–thaw cycles.

The changes in ice crystals of crayfish during FT cycles were depicted in Figure 2. The white area represented ice crystals, while the pink meshes represented muscle fibers. The myofibrils of NF crayfish were evenly distributed and compacted. The ice crystals of the three crayfish groups displayed a larger area compared to the NF crayfish with the increasing numbers of FT cycles, which was due to the irreversible destruction of cells caused by the formation of large ice crystals and recrystallization. Compared to NF crayfish, the −20 °C AF, −50 °C AF, and LNF groups presented obvious increased regions of intracellular and/or extracellular ice crystals from the 2 FT cycle, the 3 FT cycle, and the 4 FT cycle, respectively. Moreover, the ice crystals of LNF crayfish were smaller, regular in shape and evenly arranged during FT cycles compared to those of AF crayfish. Generally, the formation of ice crystals consists of two stages: nucleation and growth. Water in crayfish muscle was rapidly converted into a large number of nucleates by LNF owing to the extremely fast freezing rates, forming substantial small ice crystals. There were fewer ice nuclei formed in AF crayfish, then liquid adhered to the initial ice nucleus, resulting in larger ice crystals in muscle [45]. As shown in Figure 2B, the thawing and recrystallization resulted in the increased volume and average size of ice crystals in frozen crayfish during FT cycles. The average area of ice crystals in −20 °C AF group, −50 °C AF group, and LNF group after 5 FT cycles was 59,871 μm^2^, 42,246 μm^2^, and 41,675 μm^2^, respectively. The area of ice crystals of LNF crayfish was significantly lower than that of AF groups, demonstrating that LNF could avoid the formation of large ice crystals in crayfish muscle during 5 FT cycles. The formation of large ice crystals could squeeze and destroy the myofiber structure of crayfish during FT cycles, resulting in a wider gap between the myofibers of crayfish after thawing [23]. This observation aligned with the findings of Wan et al. [46], which indicated that FT cycles damaged the internal myofiber structure and altered the spatial conformation of MPs. Moreover, LNF crayfish had ice crystals with an elongated shape and a more compact muscle structure than AF crayfish at 5 FT cycles, indicating LNF could reduce the damage to the tissues of crayfish.

### 3.4. Microstructure Analysis

**Figure 3:**
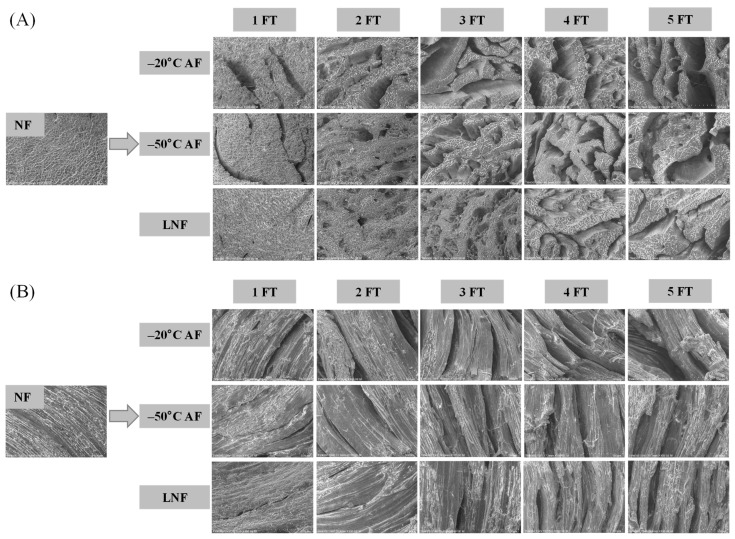
SEM images of crayfish during freeze–thaw cycles. (**A**) Transverse direction; (**B**) longitudinal direction. NF represents non-frozen crayfish, AF represents air convective freezing, and LNF represents −80 °C liquid nitrogen freezing. The labels 1, 2, 3, 4, 5 FT represent frozen crayfish with 1, 2, 3, 4, 5 freeze–thaw cycles.

The microstructure of the transverse and longitudinal direction of crayfish during FT cycles was depicted in Figure 3. The SEM images demonstrated that the muscle fibers of NF crayfish were evenly spaced and closely arranged without cracks. The gap between muscle fibers of the three crayfish groups enlarged to varying degrees with slight breakage during FT cycles. The damage and the deformation of muscle structure were mainly attributed to the formation of large and uneven ice crystals during FT cycles [25]. It can be seen that the gaps in the muscle structure of LNF crayfish were smaller than those of AF crayfish during FT cycles. This could be due to the fact that water within crayfish could quickly pass “the maximum ice crystal formation zone” by LNF, leading to the formation of small and evenly distributed ice crystals [47]. The observation of the microstructure of crayfish demonstrated that LNF could have alleviated the destruction of muscle fiber structure from expansive ice crystals and maintained the quality of crayfish during FT cycles, which was consistent with the study of Jiang et al. [48]. Moreover, the integrity of muscle tissue may contribute to better physicochemical properties, especially shear force, WHC, and DL. Dara et al. [49] have studied that MPs were the crucial components to maintain the structure of muscle fiber. Therefore, the characteristics of MPs could be further investigated to explore the effect of LNF on maintaining the quality of crayfish muscle during FT cycles.

### 3.5. Dynamic Rheological Properties of MPs

**Figure 4:**
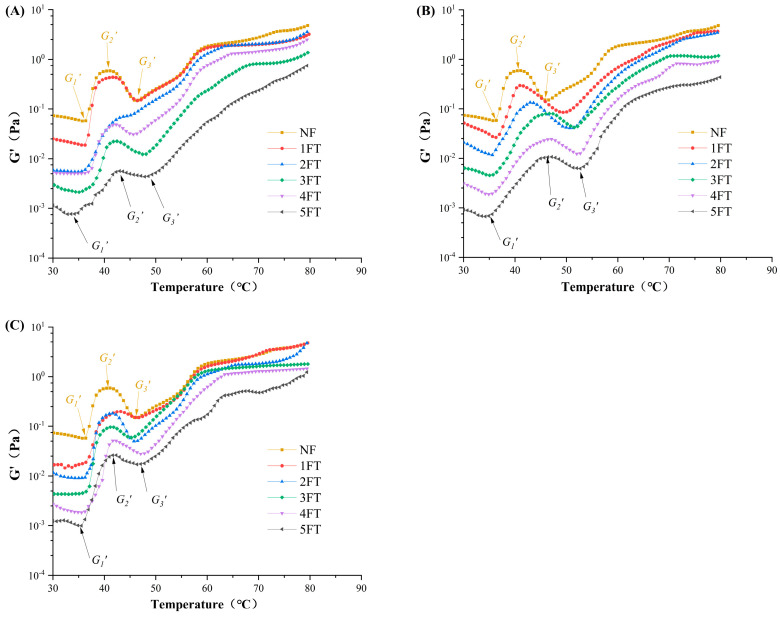
Changes in rheological properties of crayfish myofibrillar protein. (**A**) Crayfish frozen at −20 °C by air convection; (**B**) crayfish frozen at −50 °C by air convection; (**C**) crayfish frozen at −80 °C by liquid nitrogen. NF represents non-frozen crayfish. The labels 1, 2, 3, 4, 5 FT represent frozen crayfish with 1, 2, 3, 4, 5 freeze–thaw cycles.

MPs were extracted from the three crayfish groups for the analysis of changes in properties during FT cycles. The process of gelatinization indicates the protein’s capacity to form a heat-induced gel, which is associated with the physical characteristics of muscle [50]. The rheological curves of MPs from the three crayfish groups during FT cycles were shown in Figure 4. The storage modulus (*G*′) indicated the transformations occurring in protein structure, while the higher *G’* value indicated greater gel-forming ability and better sensory properties of muscle. Generally, the rheological curve of MPs can be partitioned into three main sections corresponding to the three trends of *G*′. The first stage was the formation stage of elastic gel at 30–40 °C, and the increase in *G*′_1_ was due to the unfolding of myosin filaments and the resultant linkages of myosin heads [51]. The second stage was the gel deterioration at 40–50 °C, and the decrease of *G*′_2_ value was due to the denaturation of light meromyosin. The third stage was the strengthening stage of gel at 50–80 °C, and the increase of *G*′_3_ greatly coincided with protein aggregation [52]. The declined *G′* values were due to protein denaturation, reflecting a decreased potential for gel formation [53]. This result corresponded with the research of Li et al. [11]. The variation points of the dynamic rheological curves were shown in Table 2. The highest *G*′ peak value of MPs from the three crayfish groups was decreased with the increasing numbers of FT cycles, which could be caused by the separation of actomyosin and the denaturation of myosin during FT cycles. Additionally, it might be seen that the heating temperatures of three stages in LNF crayfish (*T*_1_, *T*_2_, *T*_3_) were closer to those of NF crayfish after 5 FT cycles, suggesting LNF could better maintain the MPs structure of crayfish during FT cycles. This might be due to the formation of small and evenly distributed ice crystals in LNF crayfish, keeping hydration monolayer formed by the combination of protein and water molecules. This could alleviate the excessive aggregation of MPs and maintain protein stability [54]. For AF groups, larger ice crystals and recrystallization of crayfish during FT cycles could disrupt the connection of protein and water molecules, thus destroying the stability of MPs. This proved that LNF could better maintain the gel ability and stability of MPs during FT cycles.

### 3.6. Thermal Stability of MPs

**Figure 5:**
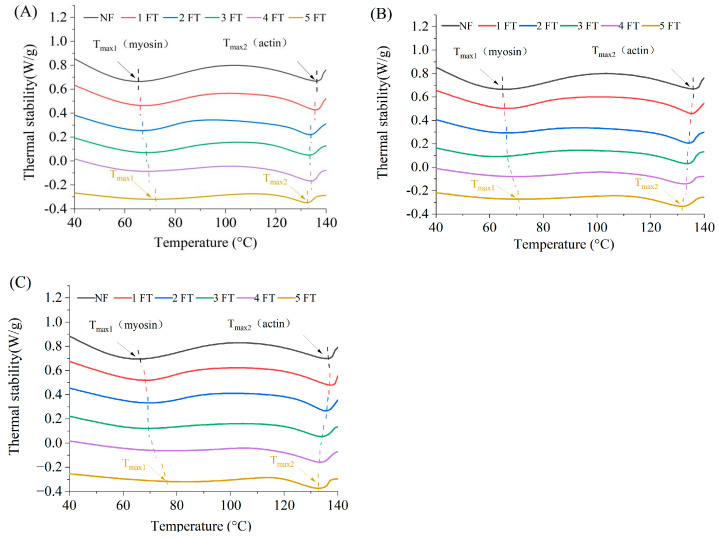
DSC curve of crayfish myofibrillar protein. (**A**) Crayfish frozen at −20 °C by air convection; (**B**) crayfish frozen at −50 °C by air convection; (**C**) crayfish frozen at −80 °C by liquid nitrogen. NF represents non-frozen crayfish. The labels 1, 2, 3, 4, 5 FT represent frozen crayfish with 1, 2, 3, 4, 5 freeze–thaw cycles.

DSC is employed to evaluate the thermal stability of MPs through the transition temperature (*T_max_*), while the enthalpy change (Δ*H*) correlates with the thermal denaturation of MPs [9]. The impact of freezing methods on the thermal stability of MPs from crayfish during FT cycles were shown in Figure 5, and the parameters were shown in Table 3. The DSC curve exhibited two distinct endothermic peaks, corresponding to myosin and actin, respectively [55]. Hydrogen bonds within myosin and actin were broken when heating, and the absorption of heat was shown as endothermic peaks, also known as denaturation peaks. Myosin had a lower denaturation temperature (*T_max_*_1_) than actin (*T_max_*_2_), indicating a lower thermal stability [56]. The *T_max_*_1_ value of NF crayfish was 65.8 °C, while that of −20 °C AF, −50 °C AF, and LNF crayfish after 5 FT cycles were increased to 67.6 °C, 68.2 °C, and 79.1 °C, respectively. Meanwhile, the *T_max_*_2_ values of these crayfish groups showed no significant difference. Wan et al. [46] studied the thermal stability of MPs of *Cyprinus carpio L.* in the FT cycle and found that the peaks corresponding to *T_max_*_1_ and *T_max_*_2_ moved to the left, and the values of *T_max_*_1_ and *T_max_*_2_ showed a significant decrease (*p* < 0.05). This proved freezing methods could significantly affect the stable spatial structure of MPs, especially myosin.

The enthalpy of myosin (Δ*H*_1_) and actin (Δ*H*_2_) of the three crayfish groups significantly decreased with the increase of FT cycle numbers. This may be a result of the protein structure unfolding and the hydrogen bond weakening during FT cycles [8]. The Δ*H*_1_ values of LNF crayfish were higher than those of AF groups before 5 FT cycles, but those of the three groups had no significant difference at 5 FT cycles. This indicates that the myosin structure in crayfish was strongly destructed after 5 FT cycles. The Δ*H*_2_ values of MPs from NF crayfish were 5.80 J/g after 5 FT cycles, and those of −20 °C AF, −50 °C AF, and LNF crayfish groups were decreased to 0.10 J/g, 1.68 J/g, and 1.92 J/g, respectively. This indicates that LNF could better preserve the structure of actin during FT cycles, thereby obtaining better thermal stability of MPs. These results demonstrate that LNF has a significant impact on the preservation of the thermal stability of MPs during FT cycles. In addition, Zhang et al. [57] have revealed that the heat-induced gelling capability of MPs plays a vital role in ensuring ideal textural properties, such as WHC and shear force of gel-type muscle food.

### 3.7. Multivariate Statistical Analysis

**Figure 6:**
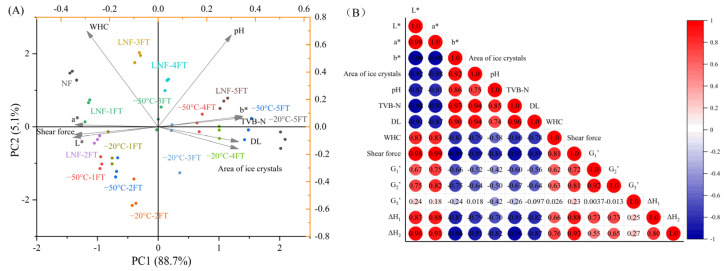
Multivariate statistical analysis of quality properties of crayfish during freeze–thaw cycles. (**A**) PCA; (**B**) Correlation heatmap. NF represents non-frozen crayfish, AF represents air convective freezing, and LNF represents −80 °C liquid nitrogen freezing. The labels 1, 2, 3, 4, 5 FT represent frozen crayfish with 1, 2, 3, 4, 5 freeze–thaw cycles.

Multivariate statistical analysis was used to collect and integrate information from various variables, aiming to reduce data dimensionality while preserving the integrity of the original data. Principal component analysis (PCA) was utilized to reduce data dimensionality while retaining the essential information. As the PCA analysis shown in Figure 6A, the first two principal components accounted for 88.7% and 5.1% of the total variance, respectively, providing a clear visualization of the relationships among the quality attributes. The PCA plot revealed that the physicochemical properties of LNF crayfish were most similar to those of NF crayfish, indicating that LNF could effectively preserve quality characteristics of crayfish during FT cycles.

The correlation coefficients of physicochemical properties and MPs properties of crayfish were constructed in Figure 6B according to Pearson correlation analysis. For two variables, a correlation coefficient (|r|) over 0.8 is indicative of a strong correlation, 0.5 to 0.8 indicates a medium correlation, 0.3 to 0.5 points to an average correlation, and anything below 0.3 is regarded as weak or uncorrelated. The correlation coefficients between *G*′_1_, *G*′_3_, and other indicators were all less than 0.8, showing a weak correlation. However, *G*′_2_ was strongly correlated with a* (r = 0.82) and shear force (r = 0.81). These results indicated that LNF mainly prevented the gel structure of MPs by inhibiting the denaturation of light meromyosin during FT cycles, thereby maintaining a better shear force of crayfish [56]. In addition, the correlation coefficient between Δ*H* and most of the indexes was greater than 0.8, indicating that LNF can maintain better physicochemical characteristics of crayfish by preventing the structure of myosin and actin during FT cycles. Among these indexes, Δ*H*_1_ was strongly correlated with TVB-N (r = −0.85), DL (r = −0.82), and shear force (r = 0.88). Δ*H*_2_ was strongly correlated with the area of ice crystals (r = −0.91), pH (r = −0.82), TVB-N (r = −0.96), DL (r = −0.87), and shear force (r = 0.93). It can be inferred that LNF may alleviate the denaturation of myosin from ice crystal damage, while myosin benefited to maintain the quality of crayfish, including the DL and shear force indexes. Du et al. [45] have proposed that the decrease in the WHC of muscle was related to the destruction of muscle fibers or myofibril structures, as well as the modification and degeneration of MPs. Moreover, the structural damage and the weakening of intramolecular hydrogen bonds of muscle would reduce the thermal enthalpy of crayfish MPs during FT cycles [23]. The formation of large ice crystals and the recrystallization during FT cycles could damage the tissue integrity, leading to decrease in the stability and dynamic flexibility of MPs [12]. These consequences resulted in a decrease in WHC of crayfish, affecting color, shear force, and DL, which in turn impacted the appearance, palatability, and processing properties of the muscle [58]. Therefore, LNF could preserve the gel strength and thermal stability of MPs by inhibiting the denaturation of myosin and alleviating destruction to the MPs structures from large ice crystals during FT cycles. The preservation of the properties of MPs and integrity of muscle structures by LNF could maintain the quality of crayfish during FT cycles.

## 4. Conclusions

This study has demonstrated that LNF is an effective method for preserving the quality of crayfish during FT cycles. The quality characteristics of crayfish deteriorated with the increasing numbers of FT cycles, including the decrease of L*, a*, WHC, and shear force, and the increase of b*, Δ*E*, pH, TVB-N, and DL. LNF crayfish could maintain better appearance and physicochemical properties during FT cycles. As observed in ice crystal morphology and muscle fiber microstructure, ice crystals in LNF crayfish were smaller and more uniformly distributed than those in AF crayfish, keeping a more compact muscle structure. Dynamic rheological analysis showed higher *G′* values and better gel-forming ability of MPs in LNF crayfish, while higher *T_max_*_1_ and lower Δ*H*_2_ from DSC results revealed the thermal stability of MPs in LNF crayfish. LNF protected the myosin structure to maintain the gel strength and thermal stability of MPs. The compact muscle structure and stable MPs properties allowed LNF crayfish to maintain quality during FT cycles. This study proposed LNF as an additive-free and eco-friendly method to maintain crayfish quality during FT cycles, providing valuable insights for the high-quality development and expansion of the industry.

## Figures and Tables

**Table 1:** Changes in quality of crayfish during freeze–thaw cycles.

Crayfish		pH	TVB-N (mg/100 g)	DL (%)	WHC (%)	Shear Force (N)
NF		7.96 ± 0.02 ^Da^	5.09 ± 0.03 ^Ea^	2.18 ± 0.14 ^Fa^	86.4 ± 0.9 ^Aa^	18.3 ± 0.4 ^Aa^
−20 °C AF	1 FT	7.91 ± 0.01 ^Ea^	8.24 ± 0.17 ^Da^	3.74 ± 0.07 ^Ea^	71.8 ± 3.1 ^Bb^	16.9 ± 0.2 ^Bb^
	2 FT	7.60 ± 0.03 ^Fa^	8.40 ± 0.13 ^Da^	5.56 ± 0.04 ^Da^	71.3 ± 2.8 ^Bb^	15.8 ± 0.1 ^Cb^
	3 FT	8.18 ± 0.01 ^Cc^	11.73 ± 0.48 ^Ca^	5.99 ± 0.23 ^Ca^	67.3 ± 4.9 ^BCb^	13.2 ± 0.2 ^Dc^
	4 FT	8.44 ± 0.01 ^Ba^	16.24 ± 0.15 ^Ba^	7.41 ± 0.15 ^Ba^	65.3 ± 1.6 ^CDb^	12.6 ± 0.2 ^Ec^
	5 FT	8.68 ± 0.04 ^Aa^	24.01 ± 0.23 ^Aa^	12.8 ± 0.22 ^Aa^	60.6 ± 0.8 ^Dc^	11.0 ± 0.1 ^Fc^
−50 °C AF	1 FT	7.69 ± 0.05 ^Eb^	7.68 ± 0.15 ^Db^	3.47 ± 0.10 ^Eb^	73.7 ± 0.3 ^Bb^	17.1 ± 0.2 ^Bb^
	2 FT	7.65 ± 0.05 ^Ea^	8.05 ± 0.04 ^Db^	4.03 ± 0.4 ^Db^	73.2 ± 1.0 ^Bb^	15.9 ± 0.1 ^Cab^
	3 FT	8.26 ± 0.04 ^Cb^	11.04 ± 0.64 ^Ca^	4.67 ± 0.26 ^Cb^	70.5 ± 1.5 ^Cb^	14.4 ± 0.2 ^Db^
	4 FT	8.38 ± 0.02 ^Bb^	15.05 ± 0.05 ^Bb^	5.46 ± 0.22 ^Bb^	67.7 ± 1.2 ^Db^	13.0 ± 0.2 ^Eb^
	5 FT	8.59 ± 0.06 ^Ab^	20.28 ± 0.48 ^Ab^	11.02 ± 0.48 ^Ab^	63.5 ± 1.0 ^Eb^	11.5 ± 0.1 ^Fb^
LNF	1 FT	7.83 ± 0.12 ^Eab^	7.61 ± 0.08 ^Db^	3.25 ± 0.04 ^Ec^	82.0 ± 0.2 ^Ba^	17.6 ± 0.1 ^Ba^
	2 FT	7.48 ± 0.01 ^Fb^	7.61 ± 0.03 ^Dc^	3.72 ± 0.09 ^CDb^	82.0 ± 0.4 ^Ba^	16.5 ± 0.4 ^Ca^
	3 FT	8.36 ± 0.04 ^Ca^	9.57 ± 0.29 ^Cb^	4.38 ± 0.58 ^BCb^	80.8 ± 0.3 ^Ca^	15.0 ± 0.1 ^Da^
	4 FT	8.29 ± 0.02 ^Cc^	12.88 ± 0.11 ^Bc^	4.96 ± 0.07 ^Bc^	75.9 ± 0.9 ^Da^	13.8 ± 0.2 ^Ea^
	5 FT	8.50 ± 0.02 ^Bb^	18.39 ± 0.77 ^Ac^	10.01 ± 0.79 ^Ab^	70.8 ± 0.7 ^Ea^	12.1 ± 0.1 ^Fa^

Notes: NF represents non-frozen crayfish, AF represents air convective freezing, and LNF represents −80 °C liquid nitrogen freezing. The labels 1, 2, 3, 4, 5 FT represent frozen crayfish with 1, 2, 3, 4, 5 freeze–thaw cycles. Different letters in the same column indicate a significant difference (*p* < 0.05). Lowercase letters indicate significant differences among freezing methods, and capital letters indicate significant differences among the numbers of freeze–thaw cycles.

**Table 2:** Rheological parameters of crayfish during freeze–thaw cycles.

Crayfish		T_1_ (°C)	*G*′_1_ (Pa).	T_2_ (°C)	*G*′_2_ (Pa)	T_3_ (°C)	*G*′_3_ (Pa)
NF		36.5	0.062	40.5	0.584	46.5	0.150
−20 °C AF	1 FT	36.2	0.019	41.7	0.439	46.3	0.150
	2 FT	36.3	0.006	43.7	0.070	63.4	1.849
	3 FT	35.0	0.002	42.4	0.023	48.2	0.013
	4 FT	36.9	0.005	42.2	0.048	45.7	0.031
	5 FT	34.3	0.001	43.1	0.006	48.6	0.004
−50 °C AF	1 FT	36.4	0.062	41.0	0.586	49.4	0.151
	2 FT	35.5	0.027	43.0	0.296	50.7	0.085
	3 FT	35.8	0.012	46.7	0.134	51.6	0.041
	4 FT	35.6	0.005	47.1	0.080	52.7	0.042
	5 FT	35.0	0.002	46.4	0.024	52.8	0.013
LNF	1 FT	33.8	0.001	43.2	0.011	46.7	0.006
	2 FT	36.3	0.061	41.7	0.584	45.7	0.151
	3 FT	35.7	0.015	41.2	0.199	45.2	0.151
	4 FT	36.2	0.010	42.4	0.181	47.8	0.050
	5 FT	35.6	0.005	41.8	0.096	47.1	0.060

Notes: NF represents non-frozen crayfish, AF represents air convective freezing, and LNF represents −80 °C liquid nitrogen freezing. The labels 1, 2, 3, 4, 5 FT represent frozen crayfish with 1, 2, 3, 4, 5 freeze–thaw cycles.

**Table 3:** Thermodynamic parameters of crayfish during freeze–thaw cycles.

Crayfish MPs		Myosin		Actin	
		*T_max_*_1_ (°C)	Δ*H*_1_ (J/g)	*T_max_*_2_ (°C)	Δ*H*_2_ (J/g)
NF		65.8 ± 0.3 ^Ea^	9.54 ± 0.04 ^Aa^	136 ± 1 ^Aa^	5.80 ± 0.02 ^Aa^
−20 °C AF	1 FT	66.9 ± 0.1 ^CDb^	8.27 ± 0.04 ^Bb^	132 ± 1 ^Ea^	5.37 ± 0.03 ^Bb^
	2 FT	66.5 ± 0.9 ^Deb^	8.13 ± 0.05 ^Ca^	133 ± 1 ^Da^	5.19 ± 0.04 ^Cb^
	3 FT	68.7 ± 0.7 ^Aa^	7.74 ± 0.04 ^Da^	133 ± 1 ^Db^	3.99 ± 0.05 ^Db^
	4 FT	68.0 ± 0.2 ^ABb^	6.74 ± 0.06 ^Eb^	135 ± 1 ^Cb^	2.34 ± 0.06 ^Eb^
	5 FT	67.6 ± 0.3 ^BCc^	5.82 ± 0.07 ^Fa^	135 ± 1 ^Bb^	0.10 ± 0.02 ^Fc^
−50 °C AF	1 FT	66.0 ± 0.1 ^Db^	8.35 ± 0.07 ^Bb^	132 ± 1 ^Da^	5.08 ± 0.03 ^Bc^
	2 FT	65.7 ± 0.1 ^Eb^	8.19 ± 0.06 ^Ca^	131 ± 1 ^Eb^	4.93 ± 0.04 ^Cc^
	3 FT	62.3 ± 0.1 ^Fb^	7.09 ± 0.03 ^Db^	135 ± 1 ^Ba^	4.92 ± 0.04 ^Ca^
	4 FT	68.2 ± 0.1 ^Cb^	6.68 ± 0.08 ^Eb^	134 ± 1 ^Cb^	1.74 ± 0.04 ^Dc^
	5 FT	68.2 ± 0.1 ^Cb^	5.81 ± 0.04 ^Fa^	136 ± 1 ^Aab^	1.68 ± 0.07 ^Db^
LNF	1 FT	69.2 ± 0.2 ^Da^	9.20 ± 0.20 ^Aa^	133 ± 1 ^Ca^	5.87 ± 0.12 ^Aa^
	2 FT	70.0 ± 3.0 ^Da^	6.90 ± 0.32 ^Cb^	133 ± 1 ^BCa^	5.48 ± 0.17 ^Ba^
	3 FT	68.1 ± 0.3 ^Da^	6.98 ± 0.19 ^Cb^	134 ± 1 ^Ba^	4.96 ± 0.10 ^Ca^
	4 FT	76.3 ± 0.4 ^Ca^	7.63 ± 0.41 ^Ba^	136 ± 1 ^Aa^	2.56 ± 0.07 ^Da^
	5 FT	79.1 ± 0.4 ^Ba^	5.82 ± 0.12 ^Da^	136 ± 1 ^Aa^	1.92 ± 0.06 ^Ea^

Notes: NF represents non-frozen crayfish, AF represents air convective freezing, and LNF represents −80 °C liquid nitrogen freezing. The labels 1, 2, 3, 4, 5 FT represent frozen crayfish with 1, 2, 3, 4, 5 freeze–thaw cycles. Different letters in the same column indicate a significant difference (*p* < 0.05). Lowercase letters indicate significant differences among freezing methods, and capital letters indicate significant differences among the numbers of freeze–thaw cycles.

## Data Availability

The original contributions presented in the study are included in the article; further inquiries can be directed to the corresponding authors.
