# Correction: Teng et al. Effect of Liquid Nitrogen Freezing on Maintaining the Quality of Crayfish During Freeze–Thaw Cycles: Muscle Structure and Myofibrillar Proteins Properties. *Foods* 2025, *14*, 279

**DOI:** 10.3390/foods14213712

**Published:** 2025-10-30

**Authors:** Zongna Teng, Xiaoyue He, Liuqing Wang, Limin Xu, Chuyi Jiao, Jiwang Chen

**Affiliations:** 1 College of Food Science and Engineering, Wuhan Polytechnic University, Wuhan 430023, China; t2806710332@163.com (Z.T.); hxiaoyue2024@163.com (X.H.); lm001011@163.com (L.X.); 2 Hubei He Yuan Gas Co., Ltd., Yichang 443000, China; 18672667710@163.com; 3 Hubei Key Laboratory for Processing and Transformation of Agricultural Products, Wuhan Polytechnic University, Wuhan 430023, China

In the original publication [1], there was a mistake in Figure 3. The authors inadvertently included two identical figures, and the SEM pictures were accidentally and wrongly exchanged during the assembly process. The corrected Figure 3 appears below. 

The authors state that the scientific conclusions are unaffected. This correction was approved by the Academic Editor. The original publication has also been updated.

## Figures and Tables

**Figure 3:**
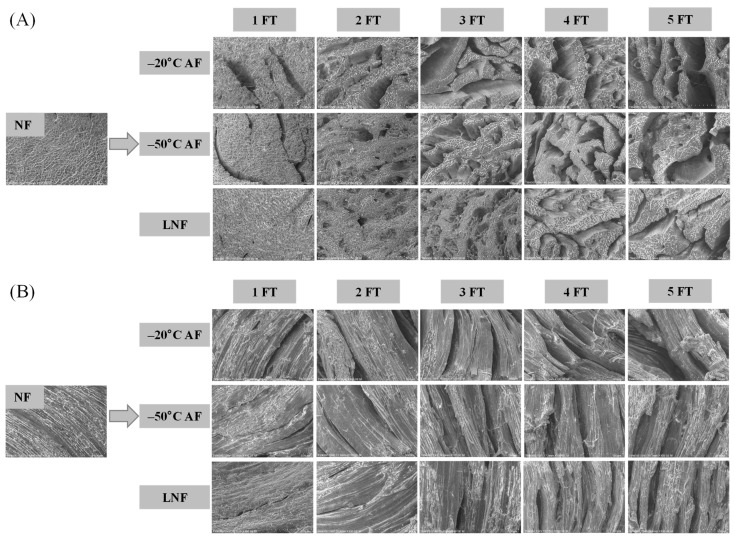
SEM images of crayfish during freeze–thaw cycles. (**A**) Transverse direction; (**B**) longitudinal direction. NF represents non-frozen crayfish, AF represents air convective freezing, and LNF represents −80 °C liquid nitrogen freezing. The labels 1, 2, 3, 4, 5 FT represent frozen crayfish with 1, 2, 3, 4, 5 freeze–thaw cycles.
